# Pregnancy-Associated Atypical Hemolytic Uremic Syndrome Successfully Treated with Ravulizumab: A Case Report

**DOI:** 10.7759/cureus.54207

**Published:** 2024-02-14

**Authors:** Yoshihiro Miyazaki, Masafumi Fukuda, Nobuhisa Hirayu, Masakazu Nabeta, Osamu Takasu

**Affiliations:** 1 Advanced Emergency and Critical Care Center, Kurume University Hospital, Kurume, JPN

**Keywords:** thrombotic microangiopathy, hemodialysis, acute kidney injury, ravulizumab, atypical hemolytic uremic syndrome

## Abstract

Pregnancy-associated atypical hemolytic-uremic syndrome (p-aHUS) refers to a pregnancy that leads to thrombotic microangiopathy (TMA). This disease is associated with adverse maternal outcomes. We encountered a case of p-aHUS, in which treatment with ravulizumab, a long-acting C5 inhibitor, resulted in a favorable clinical course and recovery of renal function. The patient was a 31-year-old woman with no apparent medical history. She developed TMA on the third postpartum day and was initially treated with steroids, plasma exchange, and hemodialysis (HD). On the seventh day of treatment initiation, she was diagnosed with p-aHUS, and treatment with ravulizumab was started. Following administration, her platelet count increased, and her acute kidney injury improved. Consequently, HD was discontinued after six sessions, and the patient was discharged on the 28th day of treatment initiation and continued her recovery at home. Similar to eculizumab, ravulizumab is an effective treatment for p-aHUS. Early administration of ravulizumab after diagnosis of p-aHUS may contribute to favorable clinical outcomes and recovery of renal function, as observed in the present case.

## Introduction

Atypical hemolytic-uremic syndrome (aHUS) is a type of thrombotic microangiopathy (TMA) caused by complement dysregulation. It is characterized by thrombocytopenia, microangiopathic hemolytic anemia, and end-organ damage [1]. aHUS is rare, with an incidence rate of 0.23-1.9 per 100,000 people [2]. However, the mortality rate in the acute phase ranges from 10% to 15%, making it a life-threatening condition [3]. The incidence of TMA during the perinatal period is oner per 25,000 births [4].

When a woman diagnosed with aHUS develops TMA during pregnancy through the postpartum period, the disease is referred to as pregnancy-associated atypical hemolytic-uremic syndrome (p-aHUS). Approximately 4% of patients diagnosed with aHUS are reported to have p-aHUS [5]. Furthermore, the majority of p-aHUS cases occur during the postpartum period, which is associated with adverse maternal outcomes [6]. The clinical course of p-aHUS is similar to that of aHUS unrelated to pregnancy, with a high likelihood of severe renal impairment, leading to end-stage renal disease in 60-70% of cases. Generally, the prognosis of p-aHUS without treatment with terminal complement inhibitors is poor. However, studies have reported successful treatment of p-aHUS using eculizumab for selective inhibition of C5 [7,8].

In this case report, we present our experience of a patient with p-aHUS, in which treatment with ravulizumab (which selectively inhibits C5 similar to eculizumab) resulted in favorable clinical outcomes and recovery of renal function.

## Case presentation

The patient was a clinically healthy 31-year-old woman with no apparent medical history. She achieved pregnancy after 15 months of fertility treatment, with successful conception occurring on the third attempt at artificial insemination. The pregnancy was uneventful, with no abnormal blood test results. Labor induction was performed at 40 weeks and five days of pregnancy as the expected delivery date was exceeded. However, it was interrupted midway, leading to a cesarean section.

**Figure 1:**
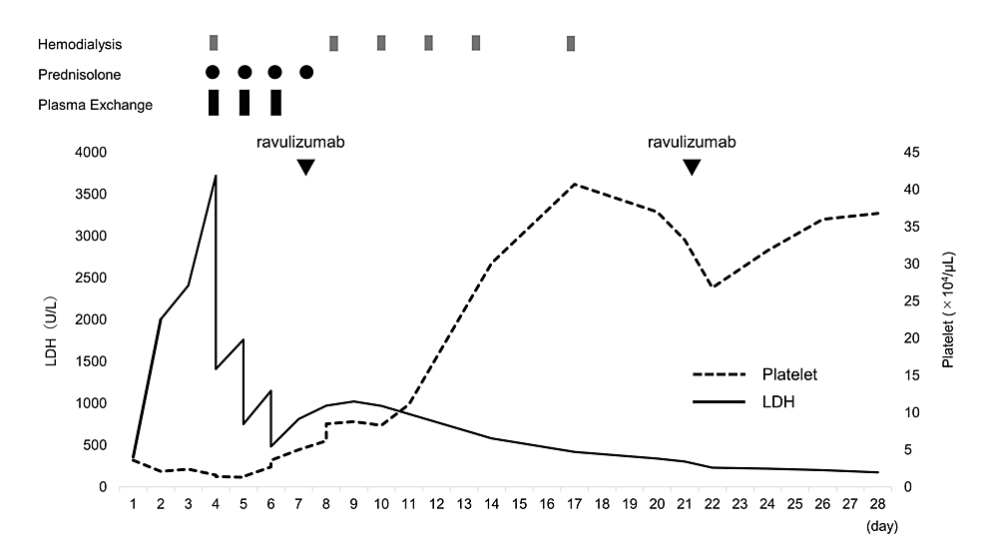
Clinical course of the patient LDH levels gradually decreased after plasma exchange, and platelet counts showed an upward trend following the administration of ravulizumab. Hemodialysis was discontinued after the sixth session on the 17th day of treatment initiation. LDH, Lactate dehydrogenase

Intraoperative blood loss amounted to 1650 mL. After an intrauterine balloon was inserted, the patient was transferred to this facility because of difficulties in controlling postoperative hemorrhage. When treatment began, the patient’s blood pressure, pulse rate, and respiratory rate were 121/80 mmHg, 136/minute, and 24/minute, respectively. Her oxygen saturation (SpO2) was 100% with the administration of 3 L of oxygen via a nasal cannula. Contrast-enhanced computed tomography revealed extravasation of the contrast agent into the uterine cavity. This finding prompted percutaneous transcatheter arterial embolization on the uterine artery to successfully control the bleeding. The clinical course following admission to the intensive care unit (ICU) is shown in Figure 1.

**Figure 2:**
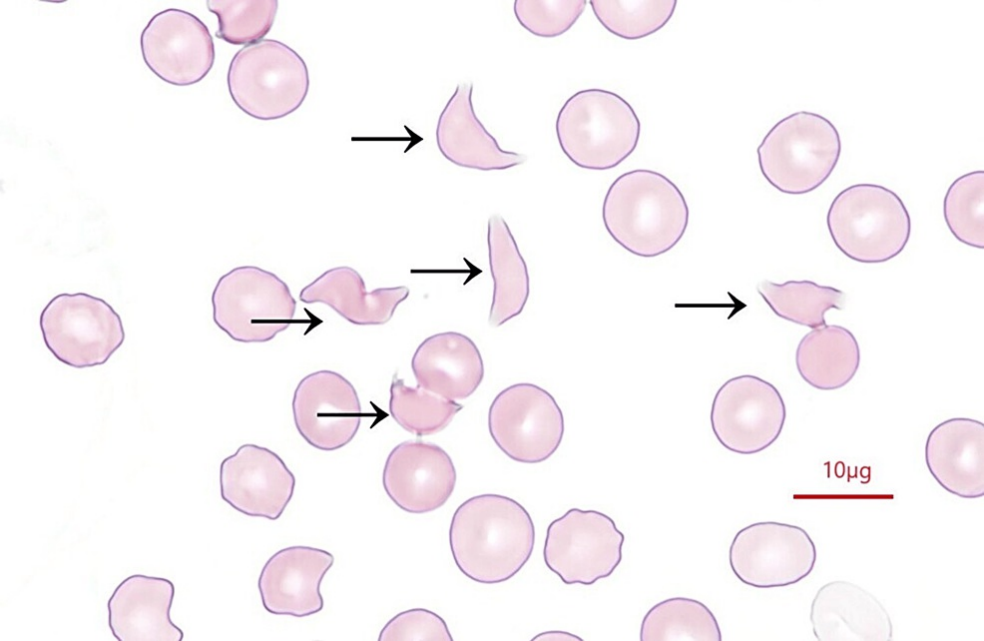
Results of peripheral blood smear examination The arrows indicate fragmented red blood cells.

Despite the absence of hemodynamic deterioration and successful control of bleeding, progressive anemia, decreased platelet count, and oliguric acute kidney injury were observed from the day following admission to the ICU. The persistent elevation of lactate dehydrogenase levels on the third day of ICU admission raised suspicion of hemolytic anemia associated with TMA. Subsequently, a peripheral blood smear examination (Figure 2) revealed the presence of fragmented red blood cells, confirming the diagnosis of TMA. The results of blood tests on the third day of treatment initiation are presented in Table 1.

**Table 1:** Laboratory data after three days of treatment PT, prothrombin percentage activity; INR, International normalized ratio; APTT, activated partial thromboplastin time; FDP, fibrin degradation products; AT3, antithrombin 3; AST, aspartate aminotransferase; ALT, alanine aminotransferase; ALP, alkaline phosphatasetotal; γ₋GTP, gamma-glutamyl transpeptidase; BUN, blood urea nitrogen; CRP, c-reactive protein; LDH, lactate dehydrogenase; ADAMTS13, a disintegrin-like and metalloproteinase with thrombospondin type 1 motifs 13

Biochemical analysis	Results	Reference range
Blood cell count		
White blood cells	15,200 /μL	3,300-8,600 /µL
Red blood cells	1.74 × 10^6^/μL	4.35 × 10^6^ to 4.92 × 10^6^/µL
Hemoglobin	5.4 g/dL	11.6-14.8 g/dL
Hematocrit	15.20%	35.1-44.4%
Platelet	1.4 × 10^4^/µL	15.8 × 10^4^ to 34.8 × 10^4^/µL
Coagulation		
PT activity	106%	80-120%
PT-INR	0.97	0.85-1.15
APTT	31.3 second	24.0-39.0 second
Fibrinogen	465 mg/dL	200-400 mg/dL
D-dimer	3.7 µg/mL	<1.0 µg/mL
FDP	16.7 µg/mL	<5.0 µg/mL
AT3	98%	80-130%
Biochemistry		
Sodium	129 mEq/L	138-145 mEq/L
Potassium	4.6 mEq/L	3.6-4.8 mEq/L
Chloride	99 mEq/L	101-108 mEq/L
AST	264 IU/L	13-30 IU/L
ALT	55 IU/L	10-30 IU/L
ALP	96 U/L	106-322 U/L
γ₋GTP	11 U/L	13-64 U/L
Amylase	231 U/L	44-132 U/L
Total bilirubin	1.5 mg/dL	0.4-1.2 mg/dL
Direct bilirubin	0.4 mg/dL	0.1-0.6 mg/dL
BUN	60 mg/dL	8-20 mg/dL
Creatinine	7.08 mg/dL	0.65-1.07 mg/dL
CRP	23.98 mg/dL	<0.14 mg/dL
Lactic acid	7.08 mmol/L	0.56-1.39 mmol/L
LDH	3,538 U/L	120-220 U/L
Additional tests for differential diagnosis		
ADAMTS13 activity	64%	>10 %
ADAMTS13 inhibitor	<0.5 BU/mL	<0.5 BU/mL
Anticardiolipin antibody (IgG)	<8 U/mL	<10 U/mL
Anticardiolipin β2GP	<1.2 U/mL	<3.5 U/mL
Antinuclear antibody	Negative	Negative
C3	68 mg/dL	73-138 mg/dL
C4	14 mg/dL	11-31 mg/dL
CH50	36.6 U/mL	25-48 U/mL
Haptoglobin	<10 mg/dL	19-170 mg/dL
Direct Coombs test	Negative	Negative
Stool culture (Enterohemorrhagic *Escherichia coli*)	Negative	Negative

While pursuing differential diagnoses of the underlying cause of TMA, steroid therapy (prednisolone 60 mg/day for four days), plasma exchange (PE), and hemodialysis (HD) were initiated. To differentiate TMA, laboratory tests were conducted to exclude conditions such as thrombotic thrombocytopenic purpura, Shiga toxin-producing *Escherichia coli*-associated HUS, autoimmune diseases, and other conditions apart from aHUS. Additionally, the absence of hypertensive disorders during pregnancy and the lack of abnormally high blood pressure at the onset of TMA led us to rule out secondary TMA, such as malignant hypertension and HELLP (hemolysis, elevated liver enzymes, and low platelets) syndrome. The diagnosis of p-aHUS was established based on these tests.

The definitive diagnosis of p-aHUS was established after three PE sessions. Consequently, treatment with ravulizumab, a long-acting C5 inhibitor, was initiated on the seventh day of ICU admission (initial dose, 2,700 mg; subsequent doses, 3,300 mg). After the initiation of ravulizumab treatment, the patient’s platelet count increased, with 6.2 × 10^4^/μL on the day after the first dose, 8.3 × 10^4^/μL on the third day of administration, and 30 × 10^4^/μL on the seventh day of administration. Additionally, her urine output increased, and six HD sessions were performed because of the rapid reduction of blood urea nitrogen and creatinine levels. Eventually, the patient was weaned off HD and discharged home on the 28th day of treatment.

After being discharged, p-aHUS treatment continued as an outpatient. Ravulizumab was administered three times, with each dose given every eight weeks as an outpatient. No recurrences were observed after treatment was discontinued. The results of a genetic test, which had been submitted on the third day of treatment, became available later, but no apparent genetic mutations were observed.

## Discussion

This case demonstrated that the administration of ravulizumab increased the platelet count and improved renal function, allowing for the discontinuation of HD. Eculizumab is considered to be effective in treating aHUS. A multicenter prospective study in Europe and North America has reported its effectiveness in increasing platelet counts and improving the estimated glomerular filtration rate in aHUS cases [9]. However, this study excluded pregnancy and p-aHUS cases. Thus, the effectiveness of eculizumab in treating p-aHUS cases remains unclear.

In a study comparing p-aHUS cases treated with and without eculizumab, none of the 10 cases treated with eculizumab progressed to end-stage renal disease, whereas six of the 12 cases not treated with eculizumab developed end-stage renal disease and ultimately required renal replacement therapy [10]. This finding suggests the potential effectiveness of selective C5 inhibitors on aHUS and p-aHUS. Similar to eculizumab, ravulizumab is an approved therapy for aHUS. Compared with eculizumab, ravulizumab can rapidly and selectively inhibit C5 for an extended duration. However, a previous study found no significant difference in the treatment efficacy or effectiveness between ravulizumab and eculizumab in aHUS cases [11]. Moreover, reports on the administration of ravulizumab for p-aHUS are very limited. In Gäckler et al.’s study, among eight patients, 87.5% showed improved platelet count, LDH level, and creatinine level to certain reference values. The median recovery time was 31.5 days, with all cases discontinuing dialysis within 21 days [12]. In the present case, the time required for recovery was 21 days, and dialysis could be discontinued at 17 days, similar to Gäckler et al.’s study.

Regarding the timing of C5 inhibitor treatment initiation for aHUS cases, a previous study found that the early group (received eculizumab within seven days of hospitalization) had shorter ICU stay and decreased number of PE and HD sessions compared with the late group (received eculizumab within eight days or later) [13]. In the present case, ravulizumab was administered on the seventh day of treatment initiation. Shortly after administration, the patient’s platelet counts, LDH, and creatinine levels improved, and dialysis was discontinued. Similar to eculizumab, starting treatment with ravulizumab early after diagnosis may increase its therapeutic effectiveness. Ravulizumab has a longer half-life compared with eculizumab, leading to a lower dosing frequency. Reports on aHUS cases have shown a 32.4-35.5% reduction in healthcare costs [14], suggesting that ravulizumab could be a viable option for p-aHUS treatment from a cost perspective.

## Conclusions

Similar to eculizumab, ravulizumab is an effective treatment for p-aHUS, and initiating administration early after diagnosis may contribute to favorable clinical outcomes and recovery of renal function. However, because of the relative rarity of p-aHUS, further studies using ravulizumab to treat p-aHUS are needed to verify our findings.
